# The effect of humic acid in composition with polyacrylamide copolymers on wind and water soil erosion

**DOI:** 10.1038/s41598-025-28807-8

**Published:** 2025-12-02

**Authors:** Neila Ye. Bekturganova, Mojtaba Mirzaeian

**Affiliations:** 1 Kazakh Automobile and Road Institute Named L.Goncharov, Raiymbek Ave., 415B, Almaty, 050061 Kazakhstan; 2 School of Computing, Engineering and Physical Sciences, University of the West of Scotland, Paisley, PA1 2BE UK

**Keywords:** Structure formation, Humic acid, Polyacrylamide, Protective crusts, Soil erosion, Ecology, Ecology, Environmental sciences

## Abstract

The present study, for the first time, the optical, electrokinetic, and mechanical properties of humic acid (HA) extracted from brown coal of the Shubarkul deposit (Kazakhstan) were investigated in combination with anionic and cationic polyacrylamide copolymers. These polymers were employed as novel, environmentally friendly, and sustainably available binders (stabilizers) aimed at mitigating soil erosion caused by wind and irrigation, while also enhancing soil structural stability. This pioneering research explores the potential of regionally sourced HA in the development of HA– copolymer polyacrylamide systems for soil protection applications. The lowest mechanical strength (Pm = 22.5 kg/cm²) was observed in the sample treated with humic acid (HA). The highest value was demonstrated by the anionic copolymer polyacrylamide, which facilitates the formation of various chemical elements and physical bonds with soil particles. The compositions of HA with P(AAm-co-AA) and P(AAm-co-DADMAC) at HA concentrations of 0.01 wt% and 0.03 wt% increased the plastic strength by 1.5 and 2 times compared to using only P(AAm-co-AA). Experimental results demonstrate effective suppression of simulated wind and water erosion in soils treated with 0.4 wt% P(AAm-co-AA) and humic acid at concentrations of 0.02 wt% and 0.05 wt%. In addition, the application of these natural binders for promoting plant growth was investigated as a novel approach. The studied compositions showed a positive effect on radish growth a plant highly sensitive to aggressive environments and toxic compounds thereby confirming the environmental compatibility and potential applicability of the materials in the agricultural industry.

## Introduction

Soil erosion, a process of soil destruction, arises from factors such as water runoff, wind displacement, and unsustainable human practices. This phenomenon results in the loss of humus—the fertile layer of soil rich in nutrients crucial for agricultural productivity—and adversely impacts water quality^1,2^, energy sectors^3,4^, infrastructure, and landscapes^5,6^. Soil erosion and land degradation significantly threaten global food security, as 95% of food consumed originates from soil. Therefore, implementing sound soil management practices is vital for mitigating erosion, conserving soil resources, and promoting sustainable development to ensure global food security according to the Food and Agriculture Organization of the United Nations^7,8^. Despite a wide range of methods such as conservative tillage, carbon farming^1^, installation of physical barriers^3^, and cultivation practices^5^, no method has proved completely effective yet. The most common and proven way is land reclamation, an age-old anthropogenic practice used in both developing and developed countries around the world. Among the most extensive reclamation activities are irrigation and drainage^9,10^. Also, Climate-smart agroforestry (CSAF), an integrated strategy for sustainable rural development, is often proposed by some scholars^11–13^. Undoubtedly, CSAF - as a holistic approach to land use that considers different pathways to address interrelated issues such as sustainable farming system and practices, mitigation and adaptation to climate change - is very important, timely and necessary. In this approach, one of the key points in improving land use is the consolidation of the fertile surface soil layer by applying various chemical reagents and their complexes with surfactants, low molecular weight compounds, etc. Successful implementation of this method depends on the economic availability of chemical reagents. A sufficient number of studies have been published in the literature on the application of synthetic and natural chemical substances such as polyvinyl alcohol^14^, polyacrylamide^4,15^, polyurethane^16^, latexes^17^, polyacrylates^18^, enzymes^19^, sodium alginate^20^ and many others used as a binder - soil stabilizer *-* to mitigate soil erosion. However, one of the main disadvantages of using these chemicals is their removal with water as they are easily washed away from soil due to their high solubility in water. To overcome the solubility problem of these chemical reagents used as soil stabilizers, some researchers have used polyelectrolyte complexes (PEC) to enhance the strength and hardening properties of soil^21–23^. PECs have macromolecular structures with repeating units which can dissociate in highly charged polymeric molecules forming a positively or negatively charged polymeric chain when dissolved in water^24^. Owing to their combined macromolecular structure and high charge, they show a strong binding property. After application onto the soil surface and subsequent drying of soil, a polymer-soil coating top layer (crust) is formed, causing soil particles glued together due to electrostatic interaction of PEC macromolecules with oppositely charged domains on the surface of particles. This functionally protects the soil from wind and water erosion^25^. As environmental safety is an important factor in soil protection, nowadays natural reagents such as humic acids and humates, due to their environmentally friendly nature and their sustainable availability, are increasingly used as soil stabilizers to ensure the environmental safety of soils^26–28^. The present work investigated the structuring properties of the associates of copolymers of polyacrylamide with acrylic acid P(AAm-co-AA), and polyacrylamide with diallyldimethylammonium chloride P(AAm-co-DADMAC) with humic acid extracted from brown coal from Shubarkul deposit (Kazakhstan) for soil protection and investigated the effect of the obtained associates on radish growth as a unique approach to highlight their environmental compatibility.

Unlike traditional chemicals that are highly prone to leaching from soil, in this work the use of the associates of environmentally friendly copolymers with humic acid originated from the Shubarkul deposit (Kazakhstan) which is used as an environmentally safe binding promoter is investigated in order to reduce the cost of the soil stabilizers and make them more accessible for upscaling in the agricultural industry. Thus, the main aim of this work is to offer an original approach to the development of environmentally and sustainable binding materials used as soil stabilizers, which combines:

- economic efficiency (use of natural materials),
- environmental safety (elimination of traditional chemicals),
- high stabilities to wind and water erosions,
- stimulating effect on plant growth.

## Materials and methods

### Materials

#### Humic acid

Materials included humic acid (HA) extracted from brown coal obtained from the Shubarkul deposit (Kazakhstan) and sodium hydroxide (NaOH, analytical grade) supplied by Sigma-Aldrich (USA). Extraction experiments were performed in a batch mixing system under atmospheric pressure using aqueous sodium hydroxide as the solvent. The process temperature was varied from 20 °C to 80 °C, while the sodium hydroxide concentration in the solution ranged from 0.5 wt% to 2.0 wt%.Humic acid extraction was studied at time intervals from 15 to 60 min. Extraction efficiency was evaluated based on HA yield as a function of exctraction temperature, duration alkali concentration and coal – to – solution ratio^28^.

#### Copolymers of acrylamide

Copolymers of acrylamide were used in this study. Poly(acrylamide-co-acrylic acid), P(AAm-co-AA), with a weight-average molecular weight (Mw) of approximately 520,000 g·mol⁻¹ and a number-average molecular weight (Mn) of 150,000 g·mol⁻¹ (typical), containing 80 wt% acrylamide, was obtained from Sigma-Aldrich (USA).

Samples were used as received without further purification. Solutions were stored in sealed bottles for at least two days prior to measurement to ensure equilibration.

Additionally, poly(acrylamide-co-diallyldimethylammonium chloride), P(AAm-co-DADMAC), with a Mw of approximately 250,000 g·mol⁻¹, was used as a 10 wt% aqueous solution, also purchased from Sigma-Aldrich (USA).

#### Soil samples

To conduct laboratory investigations the soil samples were taken on the experimental field near Erokhino village in the Pskov region (Russia) with section coordinates 56.129474 north and 31.372435 east. The soil samples were collected from the upper 10 cm of the surface horizon, representing the biologically active layer most relevant to surface processes. The sample was initially air-dried, then thoroughly homogenized by grinding in a porcelain mortar using a rubber-tipped pestle to prevent contamination. After grinding, the soil was sieved through a 1 mm mesh to obtain a uniform particle fraction suitable for further analysis. An aliquot of 0.1 g of the prepared soil was transferred into a clean glass vessel, and 30 mL of bi-distilled water was added. The resulting suspension was subjected to ultrasonic treatment for 5 min using a Digital Sonifier S-250D ultrasonic disperser (Branson Ultrasonics, USA) to ensure effective disaggregation of soil particles. The particle size distribution was determined using laser diffraction analysis with a Mastersizer 3000E laser particle sizer (Malvern Instruments, UK)^29^.

### Instrumental research

The presence of active functional groups in humic acid was determined by an FTIR spectrometer «Avatar 370 CsI» (USA) in the spectral range of 4000 –400 cm^-1^. The experimental setup included a Transmission Electronic Stability Program attachment. The samples for FTIR analysis were prepared by grinding 2 mg of the humic acid solid sample with 200 mg of KBr and then pressing the mixture into a pellet.

The pH of solutions and dispersions was measured using a pH-meter “Corning 340” (USA), equipped with a combined glass pH-electrode and built-in temperature sensor. The accuracy of measurements was ± 0.002 pH units.

A Shimadzu UV-mini 1240 spectrophotometer (USA) was used to select the working wavelength of the reagents. Based on the results obtained, the wavelength of λ = 600 nm was chosen for further studies due to the absence of chromoform groups in the visible region. The average hydrodynamic diameter of particles was determined by dynamic light scattering at a fixed scattering angle (90°) in a thermostatic cell on a Brookhaven Zeta Plus instrument (USA) where the diameter values were calculated using the DynaLS software. The electrophoretic mobility (EMP) of particles was determined by laser microelectrophoresis in a thermostatic cell using a Brookhaven Zeta Plus instrument (USA) and built-in software. All measurements were carried out at a temperature of 25 °C.

The concentration of quaternary amino groups in the cationic copolymer P(AAm-coDADMAC) was determined by turbidimetric titration with sodium polystyrene sulfonate (Mw = 100 kDa, Serva, Germany). In 0.1 weight% aqueous solution of cationic copolymer P(AAm-coDADMAC), the concentration of quaternary amino groups was 1.3 × 10^− 4^ (base-mol)/ L. Separation of the insoluble fraction of humic substance solution was carried out on an Eppendorf MiniSpin centrifuge (Germany) for 5 min at 14,500 rpm.

### Determination of strength

To measure the strength characteristics of polymer-soil crusts, a variant of the penetration method for determining the shear stress of dispersed material by indentation of a metal cone developed by P.A. Rebinder^30^ was used. Samples of sandy soil with polymer-soil crust formed on their surface were prepared as follows: 60 g of soil with a height of about 5 cm and a surface area about 17 cm^2^ was placed in each plastic cup. Around 6 mL of the test solution, which corresponds to about 3.5 L/m^2^, was sprinkled on the top of each soil sample. The samples were dried to constant weight in air at room temperature and 30% relative humidity for one week. In the studied samples, the polymer-primer crust layer was 2 to 12 mm thick. Then, using the scale of the metal-cone-indentation device, the depth of immersion of the cone in the polymer-soil crust on the surface of unstructured soil under the load necessary for the appearance of a crack on its surface was measured. The shear stress (or strength), P_l_, was calculated using the Rebinder’s formula^30^:

$${{\text{P}}_{\text{l}}}={{\text{K}}_{\text{a}}}\left( {{\text{F/}}{{\text{h}}^{\text{2}}}_{{\text{m}}}} \right),{\text{ g/c}}{{\text{m}}^{\text{2}}}$$

where F is the value of load taken from the opposite side of landing, g; h_m_ is the depth of well, cm; and K_α_ is a constant value depending on the landing angle.

### Definition of wind and water erosion

Petri dish was used for erosion control measurements. 60 g of each soil sample were poured into a Petri dish with a diameter of 9.4 cm in such a way that a layer of soil with a thickness of around 6–7 mm was obtained in each dish. The samples were sprayed with the test solution, dried at room temperature and tested for erosion. The weight loss of the samples was calculated after each erosion test.

The stability of the samples to wind erosion was studied by exposing them to an air flow generated by an electric hair dryer (BaBylissPRO^®^ Rapido, Italy). The air flow rate was controlled with a Testo 440 anemometer (Germany). The Petri dish with the sample was placed horizontally; the hot air flow (50 °С) was directed at an angle of 30° from a distance of 5 cm to the top of the sample for 10 min while the humidity was at 30%.

To study water resistance, water irrigation of the soil sample was carried out for 10 min from a distance of 20 cm in pulse mode using an atomizer with a pressure of 2 atmospheres and a water delivery rate of 200 mm/h. The inclination of the bowl during watering was 45°. The effluent water (about 250 mL in each experiment), and the sediment were collected in a glass vessel and centrifuged. The precipitate was washed three times with double-distilled water and dried to a constant weight. The supernatant was discarded; the product was obtained and weighed.

### Bioassay

The biocompatibility of the polymers was evaluated in a laboratory phytotest on polymer-treated soil using radish (*Raphanus sativus L.*) as the test culture. All experimental studies were conducted using radish (*Raphanus sativus L.*) seeds purchased from a local market, where they are freely and legally available as an agricultural product. The research did not involve any wild, protected, or endangered plant species, nor any field collection of plant materials from natural habitats. Therefore, no specific permits or licenses were required. All experimental procedures fully complied with institutional, national, and international regulations and guidelines governing the use of plant materials in research, including the principles of the IUCN Policy Statement on Research Involving Species at Risk of Extinction and the Convention on International Trade in Endangered Species of Wild Fauna and Flora (CITES). Each Petri dish contained 60 g of soil and 20 radish seeds, after which 30 mL of the polymer, humic acid, or their composite solutions were applied. The dishes were covered with transparent lids to prevent water evaporation. After five days, the plants were removed from the dishes, washed with water, and the average lengths of roots and shoots were measured.

All experiments were conducted with 3-fold repeats. Statistical data were treated with the Excel program. The tables represent mean values and their standard deviations, calculated with confidence interval of 0.95.

## Results and discussion

**Fig. 1:**
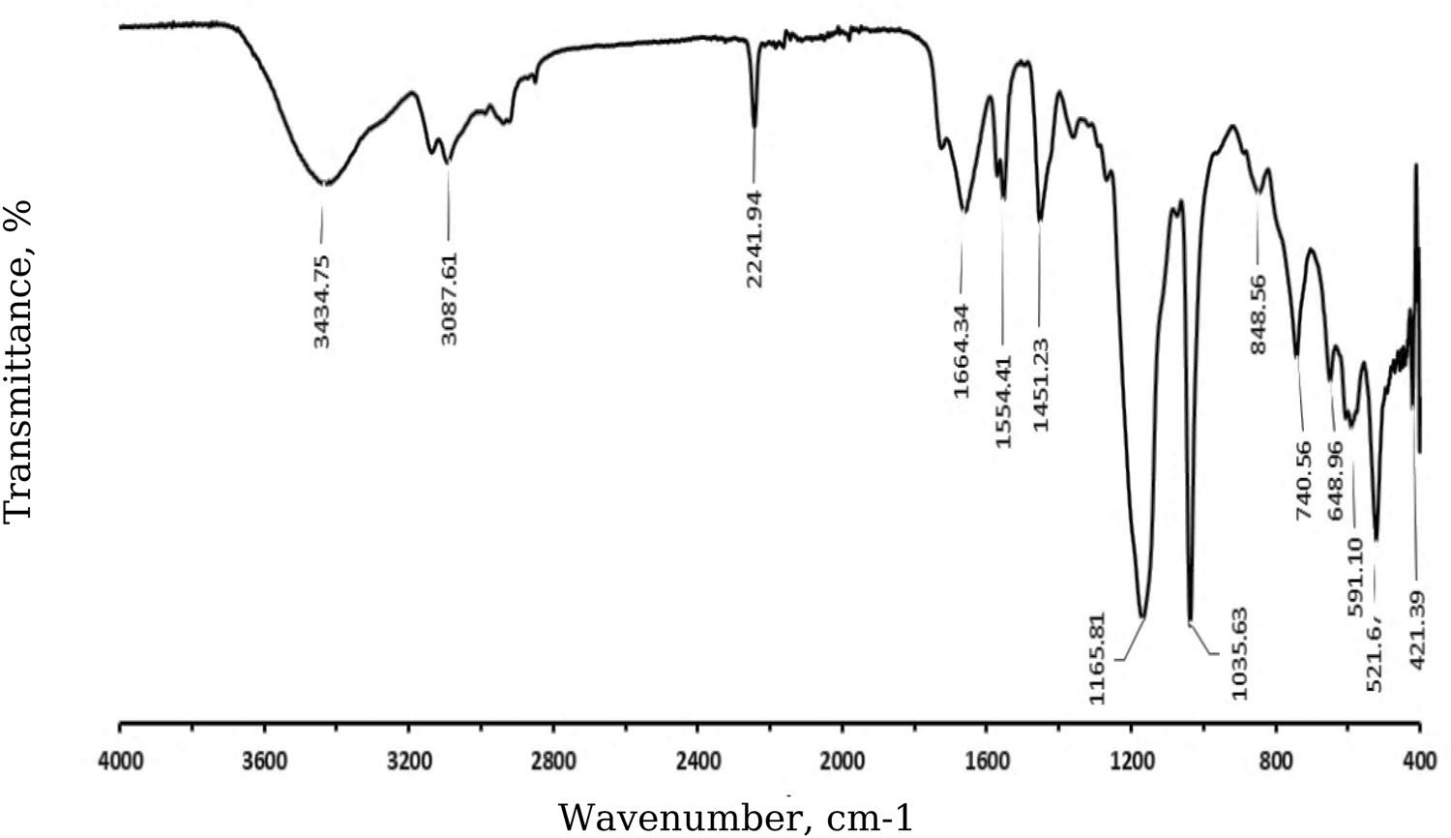
FTIR spectrum of humic acid.

The IR spectroscopic analysis provides a detailed insight into the complex molecular structure of humic acid, revealing its inherently heterogeneous and multifunctional character^31^. The spectrum distinctly indicates the presence of both aromatic and aliphatic structural domains, along with a variety of oxygen-containing functional groups, including hydroxyl (-OH), carbonyl (C = O), and ether (C–O–C) moieties (Fig. 1). These functional groups are distributed across a wide range of vibrational frequencies, confirming the chemically diverse composition of humic substances.

The broad absorption band at 3434.75 cm⁻¹ corresponds to hydroxyl groups (-OH), indicating the presence of phenolic and alcohol groups, as well as potential water content^30^.

A weak band at 3087.61 cm⁻¹ corresponds to C-H stretching vibrations in aromatic and aliphatic fragments, indicating a mixed structure^30^.

Moderate absorption at 2241.94 cm⁻^1^ is typical for disubstituted C ≡ C groups of alkynes^30^.

The strong absorption at 1664.34 cm⁻¹ is associated with carbonyl (C = O) groups, characteristic of carboxylic acids, aldehydes, or ketones^30^.

The band at 1554.41 cm⁻¹ indicates aromatic C = C vibrations, confirming the presence of aromatic ring systems within the humic acid^30^.

The deformation vibrations at 1451.23 cm⁻¹ represent C-H bonds in methyl and methylene groups, highlighting aliphatic chain components^30^.

Bands at 1165.81 cm⁻¹ and 1035.63 cm⁻¹ are linked to C-O stretching in alcohols, ethers, or esters, which further supports the presence of oxygen-containing functional groups like polysaccharides or carboxylic acids^30^.

Lower frequency bands, including 848.55 cm⁻¹, 740.56 cm⁻¹, 648.96 cm⁻¹, and others, reflect deformation vibrations in aromatic C-H bonds and additional aromatic system vibrations^30^.

Such molecular diversity highlights the highly reactive nature of humic acid, enabling it to engage in numerous physicochemical interactions within the environment. These interactions are central to its ecological functionality, particularly in the context of soil systems. Specifically, humic acids play a vital role in soil aggregation and stabilization through their ability to form complexes with metal ions and mineral surfaces. Moreover, their capacity to chelate essential nutrients facilitates nutrient retention and availability for plant uptake, thereby contributing to soil fertility and productivity. In addition, humic substances exhibit a strong affinity for various organic and inorganic pollutants, aiding in the immobilization and reduction of contaminant mobility within soil and water systems.

Taken together, the spectral characteristics observed in the IR analysis underscore the fundamental role of humic acids as dynamic and multifunctional components in biogeochemical processes, particularly in the context of sustainable soil management and environmental remediation strategies.

The granulometric composition of soil a quantitative measure of the distribution of particle sizes within a soil sample is a fundamental characteristic that directly influences numerous physicochemical and mechanical properties of the soil matrix. This parameter plays a critical role in determining the nature of interactions between soil particle surfaces and exogenous polymeric substances. In particular, the size and distribution of particles govern the degree of contact and adhesion between polymers and soil aggregates, thereby affecting the efficiency of polymer-soil composite formation and the development of protective or functional polymer-soil coatings.

Moreover, the granulometric profile is intrinsically linked to key agronomic properties of soils, such as water retention, permeability, aeration, and nutrient availability. The relative proportions of sand, silt, and clay fractions determine the total porosity and pore size distribution within the soil, which in turn controls the amount of open or void space between particles. These pore spaces serve as conduits for water infiltration and movement, and their dimensions influence capillary action, hydraulic conductivity, and the soil’s ability to retain moisture over time^32,33^.

**Fig. 2:**
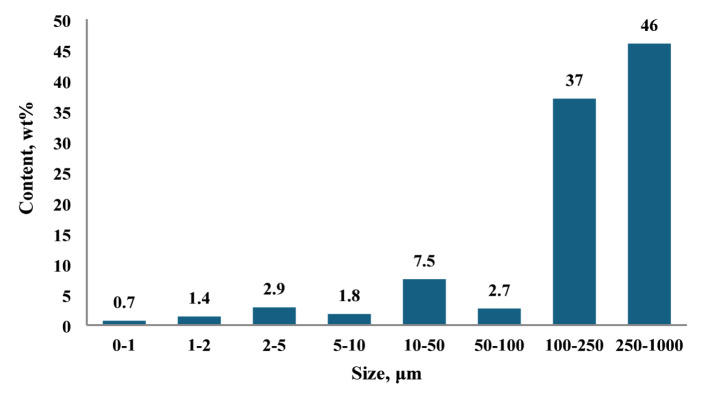
Granulometric composition of the soil sample.

The granulometric analysis of the investigated soil sample is illustrated in Fig. 2. As depicted, the particle size distribution is predominantly skewed towards the 250–1000 μm fraction, followed by the 100–250 μm fraction. Together, these two size classes constitute more than 83% of the total soil mass, indicating a substantial dominance of coarse particles. In contrast, finer particles with diameters below 50 μm account for less than 15% of the total composition.

This distribution suggests that the soil falls under the category of fine earth with a high sand content and relatively low clay fraction. Such a texture typically correlates with low water-holding capacity and limited nutrient retention potential, due to the minimal presence of colloidal clay particles which are primarily responsible for cation exchange and moisture adsorption in soil systems. Consequently, soils with this granulometric profile are often characterized by rapid drainage and low fertility, unless amended with organic matter or fine-textured materials to improve their physical and chemical properties.

The results shown in Table 1 highlights significant differences in the physicochemical properties of the studied reagent solutions, including their pH, optical density (D), electrophoretic mobility (EMP), and zeta potential (Zp). These differences provide insight into the behavior and characteristics of the solutions.

**Table 1:** pH, optical density, EMP and zeta potential data of the studied reagent solutions.

Reagent	рН	Optical density D, nm	EMP, (µm/с)/(V/cm)	Zeta potential, Zp, mV
Aqueous 0.015 wt% HA solution	3.7	450	4.81 ± 0.57	− 65.13 ± 7.77
The supernatant obtained after centrifugation of 1 wt% aqueous solution of HA	2.9	280	− 2.45 ± 0.24	− 33.26 ± 3.05
Aqueous 0.1 wt% solution P(AAm-co-DADMACl)	6.4	–	+ 4.23 ± 0.70	+ 54.19 ± 8.90
Aqueous 0.1 wt% solution P(AAm-co-AA)	5.5	–	− 2.42 ± 0.05	− 30.59 ± 0.69

In a 0.1 wt% aqueous solution of an anionic copolymer P(AAm-co-AA) containing 80 wt% acrylamide links, the concentration of carboxyl groups of acrylic acid moieties was 0.014 (basemol)/L.

The aqueous 0.015 wt% humic acid (HA) solution shows the highest optical density (450 nm), indicating a high degree of light absorption, likely due to the presence of complex organic molecules in the solution.

The supernatant obtained after the centrifugation of a 1 wt% HA solution has a significantly lower pH (2.9) and reduced optical density (280 nm), suggesting the removal of larger particles during centrifugation and an increase in acidity.

The polymer solutions exhibit neutral to mildly acidic pH levels (5.5–6.4), reflecting their distinct chemical compositions.

The aqueous 0.015 wt% HA solution has the highest negative EMP (-4.81 ± 0.57 μm/s)/(V/cm), reflecting strong repulsion among negatively charged particles, typical for humic acids.

The supernatant and polymer solutions have lower absolute EMP values, with P(AAm-co-DADMACl) showing a positive EMP (+ 4.23 ± 0.70 μm/s)/(V/cm), indicating that the positively charged species are dominant in this solution.

The zeta potential values further confirm the charge characteristics of the solutions. The HA solutions exhibit negative Zp values, with the 0.015 wt% HA solution having the most negative value (-65.13 ± 7.77 mV). This correlats well with high stability due to electrostatic repulsion.

The supernatant has a less negative Zp value (-33.26 ± 3.05 mV), likely due to the removal of larger negatively charged particles during centrifugation.

The P(AAm-co-DADMACl) solution shows a strongly positive Zp (+ 54.19 ± 8.90 mV), while the P(AAm-co-AA) solution has a moderately negative Zp value (-30.59 ± 0.69 mV), consistent with their respective polymer charges.

HA solutions are characterized by pronounced negative surface charges and high colloidal stability, attributes that are largely governed by their intrinsic molecular architecture and the abundance of ionizable functional groups, particularly carboxylic and phenolic moieties. The degree of dissociation of these acidic groups under varying pH conditions contributes significantly to the electrostatic repulsion between humic particles, thereby enhancing dispersion stability in aqueous media.

Experimental results indicate that centrifugation induces notable modifications in the physicochemical properties of humic acid solutions. Specifically, the process leads to a measurable reduction in both particle size and surface charge density, which can be attributed to the removal or sedimentation of larger colloidal aggregates and less soluble fractions. This alteration in colloidal characteristics may, in turn, influence the reactivity, mobility, and binding behavior of humic substances in environmental systems.

In contrast, the behavior of the synthetic polymer solutions reveals distinct electrochemical properties, arising from differences in their chemical composition and functional group distribution. The copolymer P(AAm-co-DADMACl), which incorporates cationic diallyldimethylammonium chloride units, exhibits a strong net positive charge in solution. Conversely, the copolymer P(AAm-co-AA), which contains weakly acidic acrylic acid residues, maintains a modest negative surface charge under neutral to slightly basic conditions.

These contrasting charge behaviors among humic and polymeric solutions highlight the broad spectrum of electrochemical interactions that can be exploited in practical applications. The diverse surface charge profiles and stability characteristics suggest tailored utility in various domains, such as environmental remediation (e.g., contaminant sorption and transport), wastewater treatment (e.g., coagulation and flocculation processes), and soil stabilization (e.g., aggregate formation and erosion control). Collectively, these findings provide a foundational understanding of the electrokinetic properties of these systems, informing their selection and optimization in applied environmental and geotechnical contexts.

To minimize environmental impact during chemical soil stabilization, environmentally safe reagents should be employed. Humic acids and their derivatives can be referred to as such substances. The advantages of HAs and humates including the retention of nutrients and water^34^, increased nutrient availability^35^, increased yield^36^, improved soil structure and reduced soil erosion^36,37^ have been widely studied. As known, HA is a complex mixture of natural compounds, whose main framework consists of an aromatic carbon skeleton substituted with functional groups. The substituents can be hydroxyl, carboxyl, alkyl, and other groups. The peripheral component is formed by polypeptide and polysaccharide fragments^25^. The presence of such a wide range of different groups in the composition of humic acids allows them to enter into ionic, donor-acceptor, hydrophobic, and other types of interactions, contributing to the binding of various classes of organic and inorganic substances. However, the bonds formed by HAs and their derivatives with soil particles are insufficient to create a strong surface crust on soil and prevent its wind and water erosion due to good HA solubility in water. To address this limitation, polymers are usually used to enhance the anchoring effect of humic substances.

In this study, commercially available polyacrylamide-based copolymers with known structuring and flocculating properties were selected for evaluating their interactions with humic acid (HA). The investigation focused on the electrochemical and colloidal behavior of mixtures formed between humic acid and polyacrylamide copolymers, particularly those bearing cationic functional groups.

**Fig. 3:**
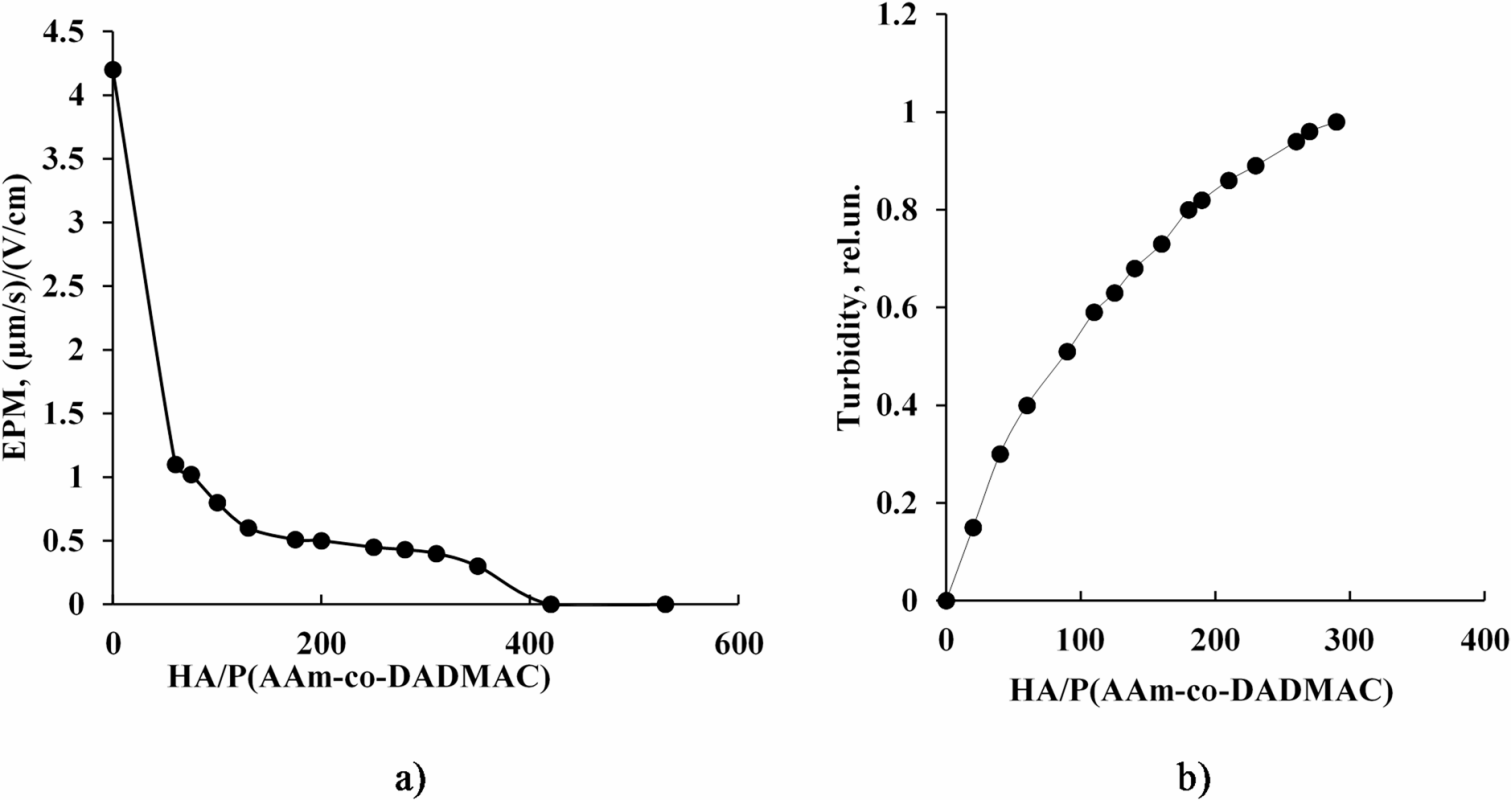
Dependences of (**a**) electrophoretic mobility of particles (EMP) in solution and (**b**) relative turbidity of solution (λ = 600 nm) when the supernatant obtained after centrifugation of 1 wt % aqueous solution of HA was added to 0.05 wt% aqueous solution of P(AAm-co-DADMAC). HA/P(AAm-co-DADMAC) is the ratio of the amount of HA (mL) to the amount of cationic copolymer (g) in solution.

The experimental results demonstrated that the gradual addition of humic acid solution (measured in mL) to an aqueous solution of the cationic copolymer P(AAm-co-DADMAC) (measured in grams) resulted in a systematic decrease in the magnitude of the copolymer’s surface charge (Fig. 3a). This trend is attributed to electrostatic interactions between the negatively charged functional groups of HA macromolecules—primarily carboxylates and phenolates—and the quaternary ammonium groups present in the DADMAC units of the copolymer. The complexation between oppositely charged species reduces the net positive surface charge of the copolymer, effectively neutralizing its electrokinetic potential.

Concurrently, an increase in the turbidity of the solution was observed (Fig. 3b), indicating the formation of larger aggregates within the system. This turbidity increase corresponds to the onset of particle aggregation, likely driven by charge neutralization and the resulting reduction in electrostatic repulsion between polymer chains and HA macromolecules.

Once the positive charges on the cationic copolymer are fully neutralized by the excess of negatively charged HA, no further increase in turbidity is detected (Fig. 3b). At this stage, the system reaches a colloidal equilibrium in which stable HA/P(AAm-co-DADMAC) complexes or associates remain dispersed in solution. These findings suggest the formation of stable polymer–humic acid aggregates governed primarily by electrostatic interactions and potentially stabilized by secondary forces such as hydrogen bonding and hydrophobic interactions.

This behavior is of particular interest for applications involving controlled aggregation, pollutant binding, or the modification of colloidal stability in environmental or industrial systems.

**Fig. 4:**
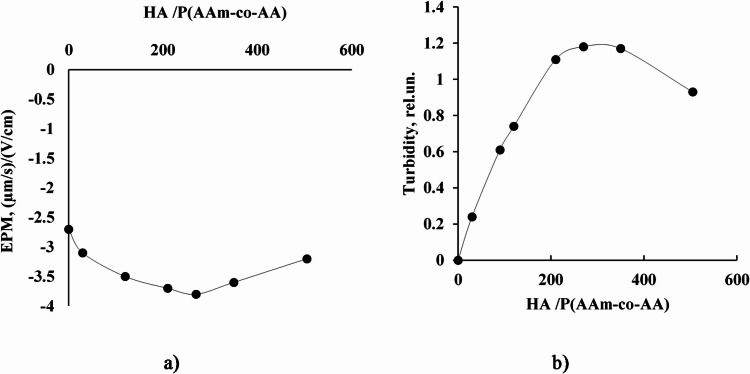
Dependences of (**a**) electrophoretic mobility of particles (EMP) in solution and (**b**) relative turbidity of solution when adding the supernatant obtained after centrifugation of 1 wt% aqueous solution of HA to 0.05 wt% aqueous solution of P(AAm-co-AA). HA/P(AAm-co-AA) is the ratio of the amount of HA (mL) to the amount of anionic copolymer (g) in solution.

In the case of the anionic copolymer system, specifically the HA/P(AAm-co-AA) mixture, the addition of humic acid does not result in a measurable change in the surface charge of the copolymer (Fig. 4a). This observation indicates the absence of significant electrostatic interactions between the two components, which is expected given that both humic acid and the copolymer P(AAm-co-AA) possess net negative charges under typical experimental conditions. Despite this electrostatic repulsion, a noticeable increase in the turbidity of the system is observed with increasing HA content (Fig. 4b), suggesting the occurrence of intermolecular interactions between the components.

The rise in turbidity is attributed to the formation of non-covalent complexes or associates between HA and P(AAm-co-AA), likely stabilized through hydrogen bonding. The carboxylic acid and amide functional groups present on both HA and the anionic copolymer provide multiple sites for hydrogen bond formation, facilitating molecular association even in the absence of favorable electrostatic interactions^36,37^.

However, when the concentration of humic acid exceeds approximately 350 mL HA per gram of anionic copolymer, a reversal in turbidity is observed i.e., the system becomes less turbid. This decline in turbidity is presumed to result from the reduced solubility of the increasingly hydrophobic HA/P(AAm-co-AA) associates. At higher HA-to-polymer ratios, the aggregated complexes likely reach a threshold beyond which they precipitate or form larger hydrophobic domains that are no longer stably dispersed in the aqueous phase, leading to phase separation or sedimentation.

These results highlight the role of non-electrostatic interactions—particularly hydrogen bonding and hydrophobic forces—in driving the associative behavior of humic substances with anionic polymeric systems. Understanding these interactions is crucial for optimizing the use of such materials in formulations for water treatment, soil conditioning, or controlled-release systems.

In the assessment of the erosion control efficacy of chemical soil conditioners, one of the key mechanical parameters is soil structural strength, which characterizes the material’s capacity to withstand mechanical loads without undergoing disintegration. This property is particularly critical in the context of surface soil layers, which are most vulnerable to erosion processes initiated by wind or water.

In naturally structured soils, the cohesive forces between particles within soil aggregates are typically sufficient to resist external mechanical stresses. These cohesive forces arise from a combination of physicochemical interactions, including capillary forces, organic binding agents, and mineral bridging. In contrast, unstructured or poorly aggregated soils exhibit minimal internal cohesion—especially in the air-dried state of the near-surface horizon—resulting in extremely low shear strength and high susceptibility to disintegration under mechanical disturbance.

The application of polymer-based soil amendments, particularly those involving humic acid (HA), can significantly modify the mechanical integrity of the soil surface by forming a polymer-soil crust. The effectiveness of this crust in enhancing soil strength and reducing erodibility is determined by two interrelated factors: (1) the mechanical properties of the interfaces between polymeric reagents and individual soil particles, and (2) the internal cohesion within polymer–humic acid (polymer–HA) complexes or associates formed in situ.

The quality of adhesion at the polymer–soil particle interface directly influences the ability of the modified soil matrix to resist mechanical breakdown. Simultaneously, the structural integrity of the polymer–HA associates, governed by intermolecular forces such as hydrogen bonding, van der Waals interactions, and potential cross-linking, contributes to the overall robustness of the polymeric network within the soil. Therefore, both sets of interactions must be considered when evaluating the potential of polymer-humic systems for long-term erosion control and soil stabilization.

**Fig. 5:**
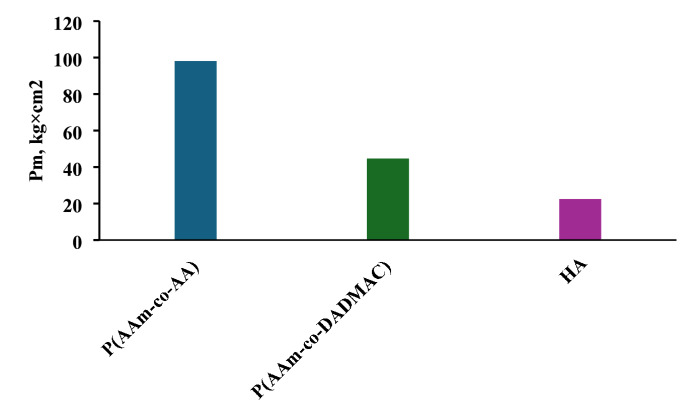
Plastic strength of soil samples treated with P(AAm-co-AA), P(Aa-co-DADMAC) and HA.

The plastic strength of soil samples treated with P(AAm-co-AA), P(Aa-co-DADMAC) and HA are shown in Fig. 5. The lowest strength is possessed by the soil sample treated with HA as Pm = 22.5 kg/cm^2^.

The enhanced mechanical strength observed in the soil sample treated with the anionic polyacrylamide copolymer can be attributed to the formation of a dense and cohesive network of intermolecular interactions between the polymer chains and the surfaces of soil particles. These interactions include multiple hydrogen bonds, electrostatic attractions, and van der Waals forces, which arise from the affinity of functional groups within the copolymer—such as carboxyl and amide moieties—for chemically complementary sites on the mineral and organic components of the soil matrix^38,39^.

This multitude of bonding interactions facilitates the effective “gluing” or binding of discrete soil particles into larger, more stable aggregates. The resulting aggregation enhances the structural coherence of the soil and significantly improves its resistance to mechanical disruption, erosion, and external environmental stresses. Such polymer-induced aggregation not only increases the mechanical integrity of the treated soil but also contributes to improved water retention and reduced surface crusting, making it particularly advantageous for applications in soil stabilization and erosion control^38,39^.

**Fig. 6:**
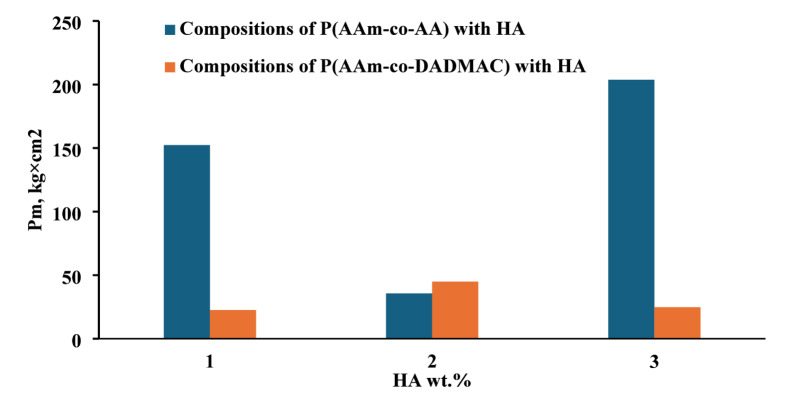
Plastic strength of soil samples treated with compositions P(AAm-co-AA) and P(AAm-co-DADMAC) with HA at different concentrations of HA 1–0.01 wt%, 2–0.02 wt%, 3–0.03 wt%.

The behaviour of cationic P(AAm-co-DADMAC) confirms the formation of associates between polymer macromolecules, HA and soil particles due to weak hydrogen and hydrophobic bonds. The effect of the addition of HA/P(AAm-co-AA) and HA/P(AAm-co-DADMAC) associates on the plastic strength of the soil crust at different HA concentrations is shown in Fig. 6. A significant increase in mechanical strength of the soil crust in comparison with its initial treatment with P(AAm-co-AA) is obtained when the soil sample is treated with the compositions of P(AAm-co-AA) with HA. Increase in the mechanical strength of soil sample treated with composition of P(AAm-co-AA) with HA for the concentrations of 0.01 wt% and 0.03 wt% HA is 1.5 and 2.0 times its initial strength when treated with P(AAm-co-AA) only (Fig. 6).

**Fig. 7:**
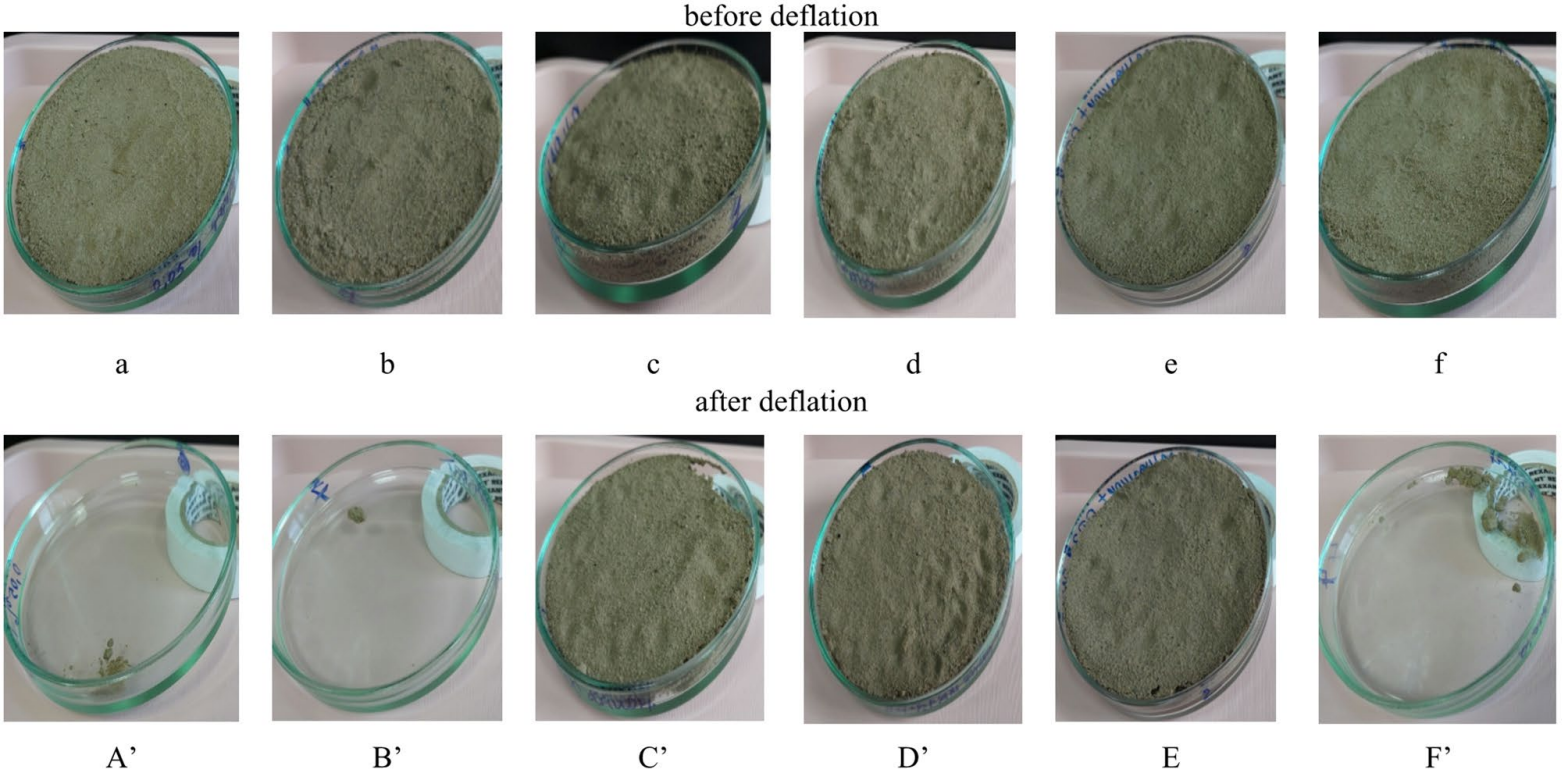
Pictures of soil samples treated with solutions of the tested reagents before (**a**–**f**) and after (**A′**–**F′**) deflation.

Subsequent research focused on assessing the influence of the investigated reagents on soil erosion resistance. The interest in the study of wind and water erosion is caused by the fact that according to available data, almost 80% of the territory of Kazakhstan is exposed to increased risk of environmental destabilization. This is due to several factors including the irrational use of land resources, intracontinental position of the country, which results in a predominantly arid climate, scarcity and uneven distribution of water resources, wide distribution of sandy and saline lands reported by the Kazinform International Information Agency^40^. In general, according to the United Nations Environment Program (UNEP) data, there is a negative trend of land cover degradation on the Earth, which requires an immediate solution to this problem^41^. The results of deflation intensity of the soil samples after sequential coating with different reagents are given in Table 2 and shown in Fig. 7. As discussed before and according to the wind erosion data given here, HA, P(AAm-co-DADMAC) and its compositions with HA (Table 2; Fig. 7a, b,f) practically do not fix the surface layer of samples, and due to which deflation intensity is high. On the contrary, the treatment of soil samples with anionic P(AAm-coAA) and its compositions with HA at consecutive application of reagents (Table 2; Fig. 7c–e) show very satisfactory and promising results with a deflation intensity of 0.1% obtained for soil sample treated by 0.4 wt% P(AAm-co-AA) / 0.05 wt% HA composition. This can be explained by the superior water absorbency of P(AAm-co-AA) polymer. In addition to its electrostatic, van der Waals and hydrogen bonds with soil particles, anionic P(AAm-co-AA) polymer is a water-absorbing hydrogel (if poor solubility and long branched chain are considered)^42,43^. When soil is treated with both P(AAm-co-AA) itself and its compositions with HA, the polymer gel granules swell rapidly. Absorption of water into the polymer molecule occurs as a result of osmosis. In this process, moisture is retained in the polymer molecule by rapid migration. Moisture-saturated polymer granules are surrounded by individual soil fractions, adhere to them and become more inert. The presence of strong bonds between aggregates contributes to the fact that the newly formed elements become stronger, heavier and more resistant to wind blowing and wear leaching^43–47^.

**Table 2:** Deflation intensity of the soil samples after sequential coating with different reagents.

Sample number	Reagent	The intensity of deflation, %
a	0.05 wt% HA	97.5
b	0.75 wt% P(AAm-сo-DADMAC)	94.8
c	0.4 wt% P(AAm-co-AA)	26.3
d	0.4 wt% P(AAm-co-AA) / 0.05 wt% HA	0.1
e	0.4 wt% P(AAm-co-AA) / 0.02 wt% HA	1.3
f	0.75 wt% P(AAm-сo-DADMAC) / 0.01 wt% HA	88.9

The evaluation of the resistance of the prepared surface coatings to water-induced erosion revealed significant differences in the effectiveness of the various treatments. In the control experiment, where the soil surface was treated with distilled water only, nearly complete leaching of soil material was observed, with soil loss approaching 100% from the Petri dish, indicating the total absence of structural stabilization.

Among the individual chemical treatments, soils treated with humic acid (HA), the cationic copolymer P(AAm-co-DADMAC), and the anionic copolymer P(AAm-co-AA) exhibited varying degrees of resistance to water erosion. The extent of soil leaching recorded for each treatment was 95 ± 5%, 86 ± 5%, and 65 ± 5%, respectively. These results suggest that P(AAm-co-AA) provides comparatively better stabilization against water erosion, likely due to the formation of more cohesive aggregates through hydrogen bonding and polymer bridging mechanisms.

It is important to note that, consistent with the findings from earlier experiments, the application of pre-mixed HA/polymer solutions to the soil surface did not result in any measurable improvement in erosion resistance. This lack of efficacy may be attributed to the formation of stable polymer–HA complexes in solution that are less reactive or less capable of interacting effectively with soil particles upon application.

Conversely, a different approach involving the sequential application of HA and polymer solutions—rather than pre-mixing—showed improved performance. The results of the water erosion tests for these sequentially applied HA/polymer treatments are presented in Table 3, demonstrating their potential for enhancing soil surface stability when reagents are applied in a controlled, stepwise manner that promotes in-situ complex formation and stronger adhesion to soil particles.

**Table 3:** Intensity of water erosion of the soil samples after sequential coating with different reagents.

Sample number	Reagent	Soil washout, %
1	0.01 wt% HA/0.75 wt% P(AAm-сo-DADMAC)	56 ± 5
2	0.02 wt% HA/0.75 wt% P(AAm-сo-DADMAC)	58 ± 5
3	0.03 wt% HA/0.75 wt% P(AAm-сo-DADMAC)	60 ± 5
4	0.04 wt% HA/0.75 wt% P(AAm-сo-DADMAC)	67 ± 5
5	0.01 wt% HA/0.4 wt% P(AAm-co-AA)	1 ± 1
6	0.02 wt% HA/0.4 wt% P(AAm-co-AA)	1 ± 1
7	0.03 wt% HA/0.4 wt% P(AAm-co-AA)	1 ± 1
8	0.04 wt% HA/0.4 wt% P(AAm-co-AA)	1 ± 1

**Fig. 8:**
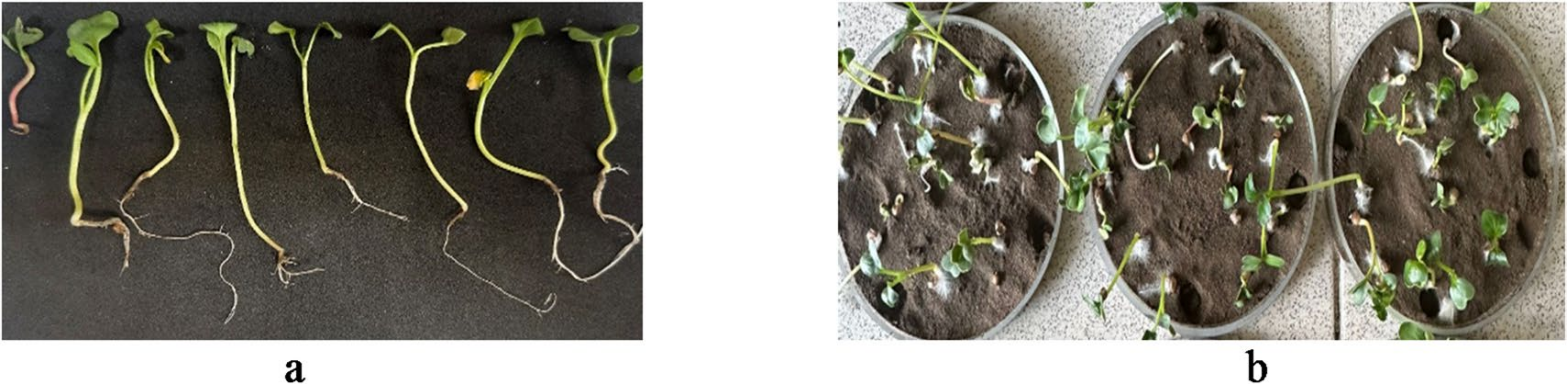
Pictures of radishes grown on soil samples treated with HA compositions with P(AAm-co-AA) (**a**) and P(AAm-co-DADMAC) (**b**) at different component ratios.

The compositions of cationic P(AAm-сo-DADMAC) polymer with 0.01–0.04 wt% HA slightly reduced soil leachability, while the compositions of anionic P(AAm-so-AA) with HA allowed a significant increase in the water resistance of the coatings by almost 100%; after sprinkling, they lost only 1 ± 1% of substrate, which correlates with wind erosion data. In addition to the surface layer fixing ability of the tested reagents, their effect on plant growth was of interest. In particular, on the growth of radish, a plant very sensitive to aggressive environments and toxic compounds (Table 4; Fig. 8). The data show that in all preparations, both cationic and anionic polymers and their compositions with HA have a stimulating effect on plant growth.

**Table 4:** Indicators of radish germination and growth.

Reagent	The number of seeds grown	Total length (stem + root), cm	Stem length, cm
Control sample	8	5	3.8
HA	10	11	6.7
0.75 wt% P(AAm-сo-DADMAC)	16	6.8	4.7
0.4 wt% P(AAm-co-AA)	19	8.6	6.5
0.4 wt% P(AAm-co-AA) + 0.05 wt% HA	15	7.6	5.8
0.4 wt% P(AAm-co-AA) + 0.02 wt% HA	16	12.6	6.3
0.75 wt% P(AAm-сo-DADMAC) + 0.01 wt% HA	15	11.6	4.8
0.75 wt% P(AAm-сo-DADMAC) + 0.05 wt% HA	19	11.5	5.5

As reported in previous studies^47,48^, humic acid is well-recognized for its plant growth–promoting properties, particularly its ability to stimulate shoot elongation and enhance overall vegetative development. These effects are largely attributed to humic acid’s influence on nutrient uptake, hormonal regulation, and improved soil structure. Additionally, polyacrylamide (PAM) and its copolymers have been documented to improve soil physical properties by increasing water-holding capacity, enhancing soil moisture retention, and promoting aggregate stability^49^. Moreover, these polymers may act as auxiliary agents that facilitate nutrient availability and retention on soil particles, indirectly contributing to improved plant health.

In the present study, the application of these reagents both individually and in combination yielded similarly positive outcomes. The observed beneficial effects likely stem from a synergistic interaction between the bioactive properties of humic acid and the soil-conditioning capabilities of the polyacrylamide-based copolymers. Together, these agents contributed to improved soil structure, enhanced moisture retention, and potentially greater nutrient availability, all of which support favorable conditions for plant growth and development.

## Conclusion

The present study systematically investigated, for the first time, the physical and mechanical properties of humic acid (HA) from brown coal of the Shubarkul deposit (Kazakhstan) in combination with polyacrylamide copolymers. This work represents a novel contribution to the field, offering new insights into the structural behavior and interaction mechanisms of HA–polymer composite systems. The research highlights the potential of these materials for applications in soil stabilization, erosion control, and environmentally sustainable technologies, particularly in the context of utilizing regionally sourced raw materials. The study demonstrates that the application of cationic and anionic polymer compositions with humic acid (HA) can significantly improve soil stability and positively influence plant growth. Specifically, the use of cationic polymer compositions with 0.01–0.04 wt% HA resulted in a slight reduction in soil leachability, while anionic P(AAm-co-AA) with HA compositions enhanced water resistance of soil coatings significantly, achieving nearly 100% stability under sprinkling conditions. This increased resistance aligns with reduced susceptibility to wind erosion.

Moreover, the tested reagents showed a notable stimulating effect on radish growth, a plant sensitive to adverse environmental conditions. All treatments, including both cationic and anionic polymers with HA, promoted higher germination rates, longer stems, and overall improved growth compared to the control sample. The highest growth indicators were observed in the sample treated with 0.4 wt% P(AAm-co-AA) + 0.02 wt% HA, yielding an average total length of 12.6 cm.

These findings highlight the dual benefits of the tested compositions in enhancing soil surface stability and supporting plant development, suggesting their potential application in agriculture and environmental management. Further studies on long-term effects and broader plant species are recommended to expand the scope of these results.

Thus, the results of this study show that the interaction of HA with both cationic and anionic copolymers of polyacrylamide forms associates stabilized by hydrogen bonds. Application of polymers, HA and their compositions on soil leads to the formation of surface crust. Soil polymer crust with the highest mechanical strength is created by compositions of P(AAm-so-AA) with HA due to high water absorption capacity of the polymer. All the studied reagents and their associations have a favorable effect on plant growth, which indicates their environmental safety. The data obtained in this work are relevant for the development of general fundamental knowledge about the properties of polymer compositions based on natural macromolecules, as well as for the practical application of binding materials. It should be noted that the study of the interaction of water-absorbing polymers with HA in the future can contribute to improving the sustainability of farming and crop production, becoming the basis for the development of new moisture-saving technologies. This will create a basis for new non-traditional methods of preservation and restoration of soil fertility, prevent environmentally adverse effects of the use of mineral fertilizers and pesticides, and reduce the manifestation of erosion processes.

## Statistics and reproducibility

All experiments were repeated independently with similar results at least three times.

## Data Availability

The datasets used and/or analyzed during the current study are available from the corresponding author upon reasonable request.

## Abbreviations

CSAF: Climate-smart agroforestry
D: Optical density
EMP: Electrophoretic mobility
FTIR: Fourier transform infrared spectroscopy
HA: Humic acid
P(AAm-co-AA): Copolymer of polyacrylamide with acrylic acid
P(AAm-co-DADMAC): Copolymer of polyacrylamide with diallyldimethylammonium chloride
Pm: Plastic strength
Zp: Zeta potential
